# Immune Evasion by the NSs Protein of Rift Valley Fever Virus: A Viral Houdini Act

**DOI:** 10.3390/v17101398

**Published:** 2025-10-21

**Authors:** Kaylee Petraccione, James G. Omichinski, Kylene Kehn-Hall

**Affiliations:** 1 Department of Biomedical Sciences and Pathobiology, Virginia-Maryland College of Veterinary Medicine, Virginia Polytechnic Institute and State University, Blacksburg, VA 24061, USA; kayleedp@vt.edu; 2 Center for Emerging, Zoonotic, and Arthropod-Borne Pathogens, Virginia Polytechnic Institute and State University, Blacksburg, VA 24061, USA; 3 Department of Biochemistry and Molecular Medicine, Université de Montréal, Montréal, QC H3T 1J4, Canada; jg.omichinski@umontreal.ca

**Keywords:** Rift Valley fever virus, NSs, immune evasion, transcription, interferon

## Abstract

Rift Valley fever virus (RVFV) is a negative-sense arbovirus that causes several severe diseases, including hemorrhagic fever in ruminants and humans. There are currently no FDA-approved vaccines or therapeutics for RVFV. The viral nonstructural protein NSs acts like a molecular Harry Houdini, the world-famous escape artist, to help the virus evade the host’s innate immune response and serves as the main virulence factor of RVFV. In this review, we discuss the molecular mechanisms by which NSs interacts with multiple factors to modulate host processes, evade the host immune response, and facilitate viral replication. The impact of NSs mutations that cause viral attenuation is also discussed. Understanding the molecular mechanisms by which NSs evades the host innate immune response is crucial for developing novel therapeutics and vaccines targeting RVFV.

## 1. Introduction to Rift Valley Fever Virus

Rift Valley fever virus (RVFV) is a highly pathogenic, mosquito-borne virus that has caused recurrent and often devastating outbreaks in both livestock and humans, with the potential to cause significant public health and economic impacts [1,2]. A member of the order *Bunyavirales* and family *Phenuiviridae*, RVFV is a negative-sense RNA virus endemic to sub-Saharan Africa and parts of the Arabian Peninsula [3]. However, its capacity to spread beyond its original epicenter, as evidenced by outbreaks in Mayotte, Yemen, and Saudi Arabia, highlights its potential for wider geographic distribution, posing an increasing risk to human and animal health worldwide [4,5,6]. First isolated in Kenya in 1931, RVFV primarily spreads through mosquito vectors such as *Aedes* and *Culex* species, but it can also be transmitted through direct contact with infected blood, tissues, or bodily fluids, as well as via inhalation of aerosolized viral particles [7,8,9].

The zoonotic and epizootic life cycle of RVFV is complex and involves transmission between mosquito vectors and vertebrate hosts [10]. Vertebrate hosts include animals such as cattle, sheep, goats, camels, African elephants, and black rhinos, with sheep and goats being particularly susceptible [11,12]. Mosquitoes amplify the virus after feeding on infected animals and transmit it to new hosts via saliva. RVFV is passed from female mosquitoes to their eggs via transovarial transmission [13]. RVFV outbreaks are closely tied to environmental conditions; for example, during periods of flooding, there is an increase in the number of hatching eggs, leading to more infected mosquitoes and allowing for transmission of the virus to new hosts [14,15].

In humans, RVFV infection presents a broad spectrum of clinical manifestations, ranging from mild, self-limiting febrile illness to more severe forms that affect approximately 10% of cases. Mild symptoms include fever, fatigue, headache, anorexia, and photophobia, whereas more severe symptoms may include ocular disease (0.5–2% of cases), encephalitis (1%), and hemorrhagic fever (1%), with mortality rates as high as 50% in cases of hemorrhagic fever [16,17]. In ruminants, RVFV outbreaks can have catastrophic consequences, with mortality rates in young ewes reaching 90–100% and nearly 100% abortion rates in impregnated animals. Such severe outbreaks can cause significant socio-economic disruptions by severely altering agriculture, trade, and livelihoods in regions dependent on livestock [18].

RVFV is classified as a BSL-3 or BSL-3/4+ agent in both the U.S. and Europe due to its ability to spread via aerosol, its high zoonotic potential, and its capacity to cause high mortality rates [19]. Increased trade and global warming have the potential to facilitate the spread of RVFV, as rising temperatures expand the geographical habitat range of mosquito vectors for this arbovirus [20]. For example, recent research has identified competent mosquito vectors and wildlife hosts in both the U.S. and Europe, including white-tailed deer [21,22,23]. There is also a continued increase in global trade, which raises the risk of infected livestock being sent to new locations and spreading RVFV to new geographic areas [24].

Given its potential as a bioweapon, RVFV is also considered a priority pathogen by the World Health Organization, a Category A priority pathogen by the U.S. National Institute of Allergy and Infectious Diseases, and a select agent by the U.S. Centers for Disease Control and Prevention and the United States Department of Agriculture [25]. Despite these classifications by various governmental agencies highlighting the importance of controlling the spread of RVFV infections, there are currently no FDA-approved vaccines or treatments. A few small-molecule compounds, such as the nucleoside analogs favipiravir, ribavirin, and 4’-fluorouridine, have shown efficacy in animal models [26,27]. Neutralizing antibodies targeting RVFV glycoproteins can protect against RVFV-induced disease in multiple animal models of infection [28,29]. A more thorough discussion of RVFV treatments is available elsewhere [30,31]. Collectively, these characteristics of RVFV highlight the critical need for research to understand its virulence mechanisms.

### 1.1. RVFV Genome Composition and Protein Functions

**Figure 1:**
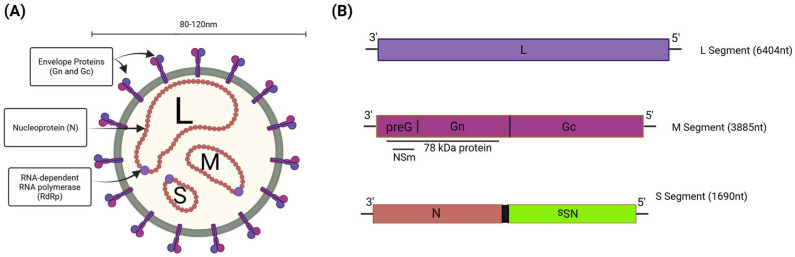
Rift Valley fever virus (RVFV) virion and genome overview. (**A**) The RVFV virion is an enveloped particle with a surface composed of glycoproteins (Gn/Gc). The viral polymerase (L, RdRp) and nucleoprotein (N) are associated with the three viral genomic segments. (**B**) The L segment encodes the L protein, the M segment encodes the NSm, 78 kDa, and Gn/Gc proteins, and the S segment encodes the N protein in the sense manner and the NSs protein in the ambisense manner. Created in BioRender. Kehn-Hall, K. (2025) https://BioRender.com/pjcaz58.

During infections, RVFV forms enveloped particles that measure 90–110 nm in diameter and possess icosahedral symmetry. The particle surface is covered by a shell of 122 glycoprotein capsomers arranged in an icosahedral lattice (Figure 1A) [32]. The viral genome is tripartite, comprising three single-stranded RNA segments: the negative-sense large (L) segment (6404 nt), the negative-sense medium (M) segment (3885 nt), and the ambisense small (S) segment (1690 nt) (Figure 1B) [33]. The L segment encodes the viral RNA-dependent RNA polymerase (L protein), which plays a critical role in viral genome replication and mRNA transcription. The M segment encodes two glycoproteins (Gn and Gc), which are important for viral attachment, fusion, and entry. The M segment also encodes the NSm protein, which has a unique antiapoptotic function and the 78 kDa protein, which is required for RVFV replication in the mosquito host [34,35]. Lastly, the S segment encodes the nucleoprotein (N) from its genomic strand, and its antigenomic strand encodes the main virulence factor, the viral nonstructural protein (NSs) [33]. The N protein is responsible for packaging the viral genome into ribonucleocapsids. Harry Houdini (1874–1926) was the world’s greatest escape artist, famous for evading seemingly impossible underwater tanks, handcuffs, and jail cells. We have coined the NSs protein as the molecular Houdini, acting to inhibit and evade the host type I interferon response, highlighting its role as the main virulence factor of RVFV [36,37,38].

### 1.2. RVFV Life Cycle

**Figure 2:**
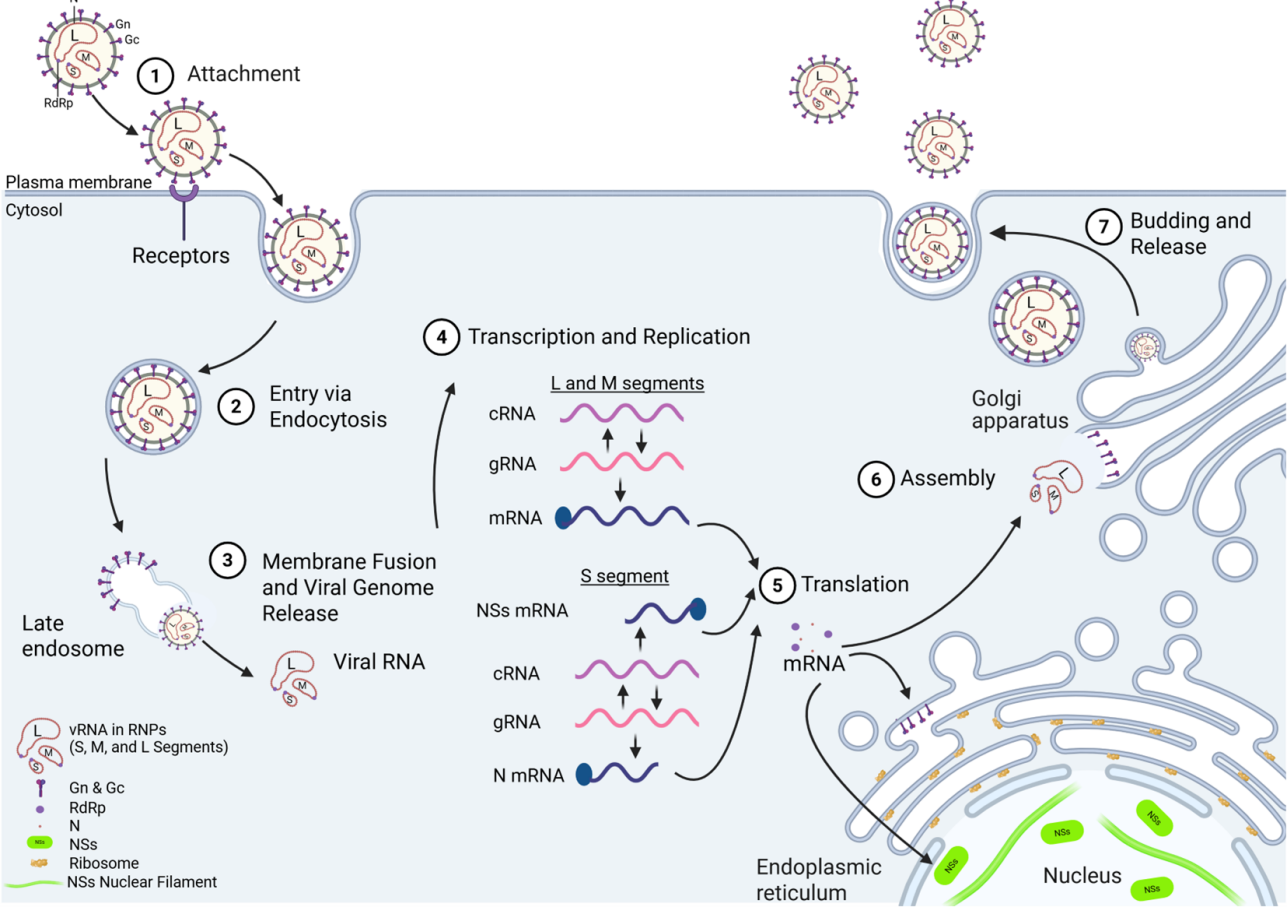
RVFV life cycle. (**1**) Attachment: Viral particles attach to the cell surface where the Gn protein interacts with host cellular receptors, including heparan sulfate, DC-SIGN, L-SIGN, and Lrp1. (**2**) Entry: Viral entry occurs through endocytosis using the host endocytic machinery. (**3**) Membrane Fusion and Viral Genome Release: In the presence of the acidic conditions in the endosome, Gc undergoes a conformational change that promotes fusion of the viral and endosomal membranes and release of the viral genome into the cytoplasm. (**4**) Replication and Transcription: The RNA-dependent RNA polymerase (RdRp) transcribes the genomic RNA (gRNA) and synthesizes the antigenomic or complementary RNA (cRNA) for replication. The gRNA serves as the template for transcription of the L, M, and N mRNAs, and the cRNA serves as the template for the NSs mRNA. (**5**) Translation: Host ribosomes translate the viral mRNAs into proteins. RdRp and N are translated in the cytosol and bind with vRNA in the Golgi apparatus for assembly. Gn and Gc are translated by membrane-bound ribosomes in the endoplasmic reticulum. The S segment antigenome (NSs-expressing) is immediately transcribed upon viral entry, and NSs localizes to the nucleus, where it antagonizes the host immune responses. (**6**) Assembly: Viral components assemble into mature particles at the Golgi apparatus and are packaged into vesicles. (**7**) Release: Viral particles bud from the Golgi apparatus, move to the cell surface in intracellular vesicles, and are released through vesicular fusion with the plasma membrane. Created in BioRender. Kehn-Hall, K. (2025) https://BioRender.com/sy5nf8e.

Replication begins with attachment of RVFV to the host cell surface, followed by its subsequent cellular entry via receptor-mediated endocytosis (Figure 2) [39]. The viral glycoprotein Gn triggers endocytosis following interaction with various cellular entry receptors, such as heparan sulfate, DC-SIGN, L-SIGN, non-muscle myosin heavy-chain IIA (NMMHC-IIA), and Lrp1 [40,41,42,43,44,45]. The low pH within endosomes leads to a conformational change in the Gc protein, mediating viral endosomal membrane fusion and uncoating, which results in the release of the viral genome into the cytoplasm [39]. Replication occurs in the cytoplasm, and approximately 10–12 h are required to complete one cycle [46]. During the initial phase of RVFV infection, the three ribonucleocapsid proteins (RNPs) containing the genome segments undergo transcription to produce the L, M, and N viral mRNAs. Next, an exact copy of the genome is replicated (complementary RNA (cRNA) or antigenome), which serves as a template for production of the new viral genome copies. The cRNA from the S ambisense segment also serves as a template for the synthesis of the mRNA for NSs. The S segment cRNA is also present at a basal level in the virion, so NSs is expressed more quickly following viral entry [47]. Transcription is initiated via the viral RdRp (L protein) performing a cap-snatching mechanism, in which host mRNAs are cleaved through the L protein’s endonuclease activity, which generates 10–18 nt-long primers for transcription of viral mRNA [48]. The mechanism by which the L protein switches between replication and transcription is unknown. However, for other bunyaviruses, elevated levels of N protein are needed for this switch to occur [49]. The L and S segment mRNAs are translated using free ribosomes, whereas the M segment mRNA is translated by endoplasmic-reticulum-membrane-bound ribosomes. Gn and Gc are processed and trafficked through the ER to the Golgi apparatus, which is the site of viral assembly. As replication continues, newly synthesized RNP segments accumulate at the Golgi apparatus, where they bind to the cytoplasmic tail of Gn [50]. Then, newly assembled virions bud into the lumen of the Golgi apparatus, where they undergo maturation followed by transport via vesicular trafficking to the cell surface and release from the cell via exocytosis [51].

## 2. NSs Protein Overview

**Figure 3:**
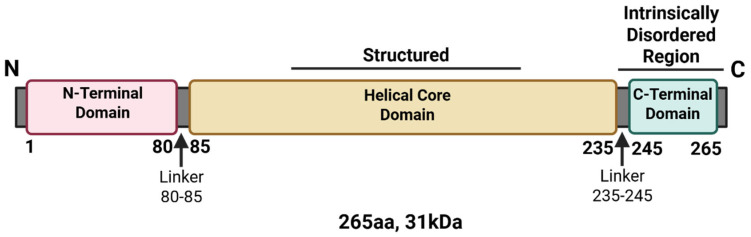
Linear structure of the Rift Valley fever virus NSs protein. The linear sequence of NSs was annotated based on termini and disordered regions. Created in BioRender. Kehn-Hall, K. (2025) https://BioRender.com/fpx9t3n.

NSs is the main virulence factor of RVFV, which is supported by studies showing that the virulent RVFV ZH548 strain causes high mortality rates in mice, whereas variant viruses carrying either partial or full deletions within NSs are avirulent and nonpathogenic in mice [36,52]. The Clone 13 vaccine strain of RVFV, in which 69% of the open reading frame of NSs is deleted, is significantly attenuated in sheep and has a 0% abortion rate in comparison to the wild-type virus, which has an ~100% abortion rate [37,53]. The NSs protein is located in both the nucleus and cytoplasm of infected cells at various times following infection with RVFV, and it forms filamentous structures in the nucleus during the early stages of infection and in the cytoplasm during later stages [54]. These filamentous structures are critical for immune evasion and modulation of host cell processes [55,56]. NSs is composed of 265 amino acids (aa) with a molecular weight (MW) of approximately 31 kDa [54]. There are three domains within NSs: the N-terminal domain (comprising 80 aa), the structured helical core region (comprising 150 aa), and the intrinsically disordered C-terminal domain (comprising 20 aa) (Figure 3). The highly acidic C-terminus has been shown to be required for the nuclear filamentous structures that NSs forms [54]. However, the helical core domain is also thought to be responsible for filament formation, as structural studies demonstrated an all-helical fold organized into highly ordered fibrils [56]. Amino acid substitutions at key residues within these domains resulted in significant loss of filamentous structures, as discussed below [54,56].

## 3. NSs Nuclear Filaments

### 3.1. Nuclear Import of NSs

NSs acts primarily in the nucleus to counteract host immune responses. However, its mechanism of transport from the cytosol into the nucleus remains unclear. At roughly 30 kDa, the MW of NSs is below the limit of 40 kDa generally considered plausible for passive diffusion through the nuclear pore. Despite this, passive diffusion is not thought to be the mechanism of NSs nuclear entry. Studies have found that nuclear import of NSs is dependent on PXXP proline-rich motifs located within the N-terminus (positions 29–32), the helical core domain (positions 82–85), and the C-terminus (positions 240–246); disruption of the PXXP motifs prevented nuclear import and filament formation [57] (Table 1). As proline-rich sequences in other nuclear-localized proteins often mediate protein–protein interactions, this suggests that NSs binds to host cell nuclear proteins through these proline-rich motifs to enter the nucleus [27]. As NSs does not contain a nuclear localization signal (NLS), it is expected that the target host protein(s) would contain an NLS. It has been shown that the nucleoporin protein Nup98 interacts with NSs, and when either Nup98 or Nup62 is depleted, the cytoplasmic fraction of NSs increases. The decrease in nuclear import of NSs was more significant upon Nup98 depletion [58,59], indicating that Nup98 contributes to NSs nuclear import. Signal transducer and activator of transcription 3 (STAT3) has also been implicated in the nuclear localization of NSs. NSs was predominantly cytoplasmic in mouse embryonic fibroblasts (MEFs) lacking STAT3, in contrast to the typical nuclear and filamentous NSs observed in wild-type MEFs. This suggests that STAT3 is also required for nuclear localization of NSs [60]. While the exact mechanism for nuclear entry of NSs has not been fully characterized, results from previous studies suggest that a combination of host factors contributes to the nuclear transport of NSs.

### 3.2. Structure and Function of NSs Filaments

The NSs protein has a unique ability to form filamentous structures in the nuclei of RVFV-infected cells, and these nuclear filaments serve as a mechanism to induce targeted and efficient degradation of host cellular proteins that promote viral replication [54,61,62]. NSs filament formation is also critical for viral replication and pathogenesis in vivo in mice [63]. The filaments are composed primarily of NSs, but several host proteins co-localize with the filaments, such as p44, XPB, SAP30, YY1, and LC3 family members. This co-localization of host nuclear proteins is thought to impede the host immune response, as further described below [63,64,65].

Filaments are detectable in the nuclei of infected cells within 4–5 h post-infection, and their presence during the early stages of infection correlates with an increased viral load [55]. NSs assembles into disulfide bond-dependent fibrillary aggregates [54,55,61], forming large filamentous aggregates within the nucleus and smaller globular aggregates in the cytosol [61]. These nuclear filaments have been shown to be the first example of amyloid-like fibril aggregates formed by a virus. Their properties include an ultrastructure of 12 nm in width, strong resistance to detergents, and the ability to interact with the amyloid-binding dye Thioflavin-S [55]. At the start of infection, NSs nuclear fibrils are parallel and straight. As infection progresses, the fibrils grow in size between 8 and 22 nm in width, and the structure and size of the late filaments are similar to the amyloid fibrils formed by the Tau protein in neurodegenerative diseases such as Huntington’s and Parkinson’s [55].

### 3.3. Amino Acid Substitutions That Alter NSs Filament Formation

Multiple studies have investigated the structural determinants of NSs filament formation. A stable construct was designed to enable expression and purification of NSs through deletion of both the N-terminal 82 residues and the C-terminal 17 residues (NSs-ΔNΔC) [54,57,66]. Viruses expressing the ΔNΔC version of the NSs protein still induced filament assembly within infected cells, suggesting that the N- and C-terminal domains are not strictly required for filament formation. However, these findings were inconsistent with a few other studies that have demonstrated that single amino acid substitutions within NSs can limit filament formation. For example, a short motif located in the C-terminus (_261_FVEV_264_) was shown to be required for the formation of NSs filaments in the nucleus (Table 1). The motif was termed the ΩXaV motif, where Ω is Trp or Phe, X is any amino acid, a is Asp or Glu, and V is Val. Substitution of the first amino acid to either Ser or Pro (F261S, F261P) resulted in abrogation of nuclear filament formation, indicating a crucial role for the C-terminus in filament formation [66].

In addition to the previously identified ΩXaV motif, a recent study examined which amino acid residues were strictly needed for NSs filament formation [63]. To this end, plasmids encoding NSs variant proteins with alanine substitutions at several sites were constructed (R16A, N153A, D157A, P240A, P80A, P81A, P82A, K84A, R88A, C178A, E221A, S228A, R30A, K150A, T168A, R173A, R235A, N237A, and R164A). Of the substitutions tested, K150A, T168A, R173A, R235A, and N237A all displayed more diffuse filament distribution but still partially formed filaments. In contrast, the R164A substitution caused the most pronounced impairment of NSs filament formation, leading to a diffuse cellular distribution of the variant NSs protein when expressed in cells (Table 1). Structural studies of NSs indicate that the R164 residue participates in the formation of four hydrogen bonds at the center of the U1-U2 dimer interface, a critical region for filament assembly. The structural studies, in combination with the substitution experiments, suggest that the R164 residue plays a critical role in stabilizing NSs dimerization and NSs filament formation. Interestingly, RVFV expressing the R164A residue variant NSs protein showed a 2 log_10_ decrease in viral replication and reduced virulence in vivo, with a fatality rate in mice of 8.3% in comparison to 100% with the wild-type NSs. These findings demonstrate that the R164 residue is essential for NSs filament formation and a key component of RVFV replication and pathogenicity [63].

**Table 1:** RVFV NSs mutations impacting its function.

**Motif or aa**	**Function (s)**	**Location in NSs**	**Variant Tested**	**Effect on Viral** **Kinetics**	**In Vivo Effect**	**References**
R16/M250	1. NSs transcription inhibition 2. p62 degradation 3. Induction of pyroptosis [67]	N-terminus: position R16, and C-terminus: position M250	R16H/M250K	~Half-log reduced replication compared to WT MP-12	1. R16H/M250K displayed 100% survival	[63,68]
PXXP	1. NSs nuclear import 2. Filament formation	N-terminus: positions 29–32 (PP1), helical core domain: positions 82–85 (PP2), and two motifs within the C-terminus: positions 240–246 (PP3/4)	PP1: ARIA PP2: AAKA PP3/4: AVIAAIA	Not tested	Not tested	[57]
K150	1. Diffuse filament distribution	Helical core domain: position K150	K150A	Not tested	Not tested	[63]
R164	1. NSs filament formation 2. TFIIH degradation	Helical core domain: position R164	R164A	2 log_10_ decrease in viral replication from in vivo tissue samples	1. R164A significantly delayed fatal infection 2. 8.3% mortality rate	[63]
T168	1. Diffuse filament distribution	Helical core domain: position T168	T168A	Not tested	Not tested	[63]
R173	1. NSs’ ability to degrade PKR 2. Diffuse filament distribution	Helical core domain: position R173	R173A	1.5 log_10_ decrease at MOI 0.01, no effect at MOI 1.0	Not tested	[63,69]
R235	1. Diffuse filament distribution	Helical core domain: position R235	R235A	Not tested	Not tested	[63]
N237	1. Diffuse filament distribution	Helical core domain: position N237	N237A	Not tested	Not tested	[63]
M250	1. NSs transcription inhibition	C-terminus: position M250	M250K	No effect	1. 20% fatality rate in M250K compared to 55% fatality in WT	[68]
F261	1. NSs filament formation 2. TFIIH-p62 degradation 3. NSs interaction with LC3 4. NSs inhibition of autophagy	C-terminus: positions: 261–264	F261S, F261P, V264S	Upon substitution (F261S, F261P) there is a 1 log_10_ decrease in HSAEC cells (MOI 3.0)	Not tested	[66,70]

## 4. NSs Prevents IFNAR Signaling and Downregulation of Cellular Transcription

### 4.1. NSs Suppression of IFN-β

**Figure 4:**
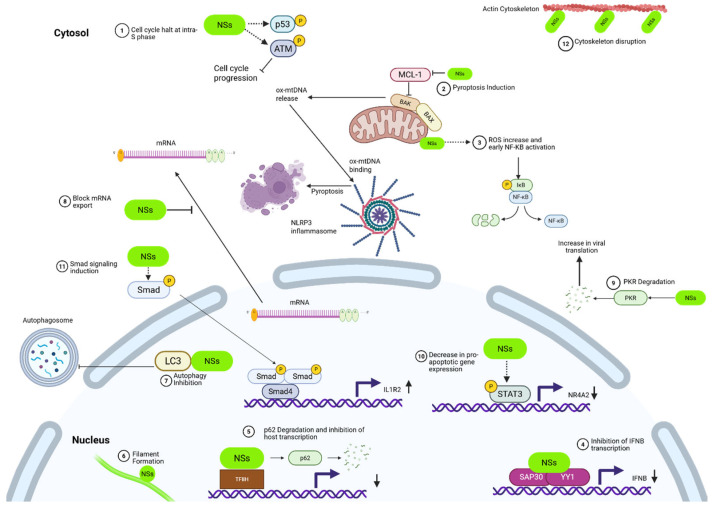
NSs cellular takeover. 1. Cell cycle inhibition: NSs induces arrest at the intra-S phase via activation of the ATM arm of the DNA damage response (DDR) while suppressing ATR signaling. 2. Pyroptosis induction: NSs promotes inflammation by downregulating the antiapoptotic protein MCL-1, enabling BAK activation, mitochondrial outer membrane permeabilization (MOMP), and accumulation of mitochondrial ROS (mtROS). This leads to cytosolic release of oxidized mitochondrial DNA (ox-mtDNA), which activates the NLRP3 inflammasome and triggers gasdermin D (GSDMD)-mediated pyroptosis in infected cells. 3. NF-κB activation: NSs localizes to the mitochondria, disrupts electron transport, and increases ROS, leading to the activation of NF-κB. 4. IFN-β promoter inhibition: NSs antagonizes type I IFN signaling by forming a corepressor complex with SAP30 and YY1. The ternary complex inhibits the IFN-β promoter and blocks activation of the innate immune response. 5. Inhibition of host transcription: NSs disrupts RNA polymerase II-mediated transcription by targeting TFIIH. NSs binds directly to the p44 and p62 subunits and indirectly to XPD, sequestering them into nuclear filaments. p62 is additionally subject to proteasomal degradation via FBXO3, which results in the inhibition of RNA polymerase II-mediated transcription. 6. Nuclear filament formation: NSs forms filamentous nuclear structures that function as hubs for targeted degradation of several host proteins, including several subunits of TFIIH, which facilitates viral immune evasion and replication. 7. Autophagy inhibition: NSs interacts with LC3 via an LIR motif (NSs4), sequestering it in the nucleus and blocking autophagosome formation in the cytosol, thereby suppressing the autophagic response during RVFV infection. 8. Inhibition of mRNA export: NSs inhibits nuclear export of host mRNAs, contributing to global suppression of host gene expression and promoting viral gene dominance. 9. PKR degradation: NSs mediates degradation of PKR, preventing host-mediated viral translation shutdown and blocking antiviral immune signaling. **10**. Modulation of apoptosis: NSs induces phosphorylation of STAT3 on a conserved tyrosine, promoting nuclear translocation of STAT3 and downregulation of proapoptotic genes such as NR4A2. STAT3 activation enhances cell survival during RVFV infection. **11.** Smad signaling induction: NSs is essential for Smad phosphorylation during active RVFV replication. Smad4 complexes bind promoters of immunosuppressive targets such as IL1R2, leading to suppression of IL-1 signaling. This shift to an anti-inflammatory profile, including IL-10 and IL1RA upregulation, contributes to immune evasion and pathogenesis. **12.** Cytoskeleton disruption: NSs localizes within actin-rich intercellular structures, suggesting a role in cytoskeletal remodeling and enhancement of viral spread between cells. Created in BioRender. Kehn-Hall, K. (2025) https://BioRender.com/4fr2abj.

Type I interferons (IFNs) play a crucial role in coordinating innate immunity during viral infection [71]. Various cells, including myeloid cells, epithelial cells, and stromal cells, are stimulated through type I IFN receptors (IFNAR), which induce several IFN-stimulated genes (ISGs) to suppress viral replication [72]. Transcriptional regulation of IFN-β requires both specific binding of transcription factors and the recruitment of chromatin-remodeling complexes to the promoter region. In eukaryotic cells, activation of tightly regulated genes, including IFN-β, depends on chromatin remodeling to allow RNA polymerase II access to DNA [73,74]. NSs is a type I IFN antagonist [57], and one mechanism by which it accomplishes this is through simultaneously interacting with the Sin3A-associated protein 30 (SAP30) and the Yin Yang 1 protein (YY1) to form a SAP30-NSs-YY1 corepressor complex on the IFN-B promoter [65,75] (Figure 4). SAP30 is a subunit of the Sin3 histone deacetylase complex, which acts as a transcriptional repressor through recruitment of a histone deacetylase [76]. YY1 is a transcription factor with the ability to both activate and repress gene expression, and it plays a critical role in several cellular processes, including apoptosis and cell growth [77]. The RVFV NSs protein induces subnuclear redistribution of chromatin-remodeling corepressor components, including SAP30, YY1, and Sin3A-associated corepressor factors, by sequestering them into the nuclear filaments to target the IFN-β gene into a repressed state. In uninfected cells, IFN-β gene expression is suppressed, which correlates with the SAP30/Sin3A/NCoR/HDAC-3 corepressor complex being present on the IFN-β promoter. Upon RVFV infection, this repressive complex (SAP30/Sin3A/NCoR/HDAC-3) is reinforced by the presence of NSs at the IFN-β promoter, and histone residues K8H4 and K14H3 are maintained in their repressed, deacetylated state [65]. NSs maintains the IFN-β promoter in a transcriptionally silent state through both the formation of a ternary complex with SAP30-YY1 and chromatin remodeling [65].

Within the core domain of NSs, amino acids 210–230 are essential for its interaction with SAP30. When this region of NSs is deleted, the resulting virus (rec-ZHΔ210–230) maintains similar viral titers as the wild-type RVFV, but the resulting NSs protein in the variant virus does not associate with SAP30-YY1 on the IFN-β promoter. In mice, infection with the rec-ZHΔ210–230 variant of RVFV results in 100% survival, demonstrating that this variant virus completely loses its in vivo virulence. In comparison, all mice infected with the wild-type RVFV died. These results demonstrate the crucial role of the NSs-SAP30-YY1 interaction and IFN-β suppression for NSs-mediated pathogenicity [65].

These mechanistic insights into IFN suppression by the NSs protein are supported by in vivo studies. The highly virulent RVFV strain ZH548 inhibits the induction of the IFN-α/β response by the host, which enables RVFV to replicate efficiently and cause significant disease in IFN-competent mice. In contrast, Clone 13, an isolate of RVFV with a large in-frame deletion of the NSs gene, is highly attenuated and immunogenic in IFN-competent mice [36,37,78], demonstrating the importance of NSs suppression of IFN-α/β for virulence. Moreover, Clone 13 is highly virulent in mice defective for the IFN-α/β response, which highlights the crucial role of the interferon pathway in the host response to an RVFV infection [36]. Taken together, these results demonstrate that NSs is an IFN-α/β antagonist in vivo and that suppression of IFN is a key factor for RVFV virulence.

### 4.2. Interaction with p44 and p62 Subunits of TFIIH to Downregulate Cellular Transcription

One of RVFV’s main immune evasion strategies is the NSs-dependent inhibition of host transcription, and, as discussed above, this is linked to its unique ability to form nuclear filaments. One of the key targets of nuclear filament formation appears to be the host general transcription factor IIH (TFIIH). [64]. TFIIH is composed of 10 subunits that can be divided into two subcomplexes: (i) the core subcomplex, consisting of XPB, XPD, p52, p44, p62, p34, and p8, and (ii) the cyclin-activating kinase (CAK) subcomplex, consisting of cdk7, cyclin H, and MAT1. TFIIH is required for the formation of the transcription preinitiation complex, which is a key step required for RNA polymerase II-mediated transcription [79]. Following RVFV infection, NSs enters the nucleus and prevents the assembly of TFIIH through direct interactions with both the p44 and p62 subunits. Through these interactions, NSs sequesters the p44, p62, and XPD subunits in the NSs-generated nuclear filaments. Once sequestered in the nuclear filaments, the p62 subunit is rapidly degraded in a ubiquitin–proteasome-dependent manner [64,68,80]. The ability of NSs to degrade p62 has been shown to be dependent on an ΩXaV motif in the C-terminal region of NSs [66]. This degradation occurs through NSs also sequestering the ubiquitin ligase FBXO3 within nuclear filaments [63,81]. The key role of FBXO3-dependent degradation of p62 during RVFV infection is supported by experiments demonstrating that knockdown of FBXO3 partially rescued NSs-induced IFN suppression [81] and that inhibitors of the ubiquitin–proteasome system stabilize p62 levels in RVFV-infected cells. Importantly, loss of p62 was shown not to be a result of either transcription inhibition or mRNA stability [80].

Although NSs binding to p62 through its ΩXaV motif has been shown to be important for nuclear filament formation, several regions within the core domain have also been shown to be crucial for filament formation and thus NSs-mediated inhibition of host transcription (Table 1) [63]. In particular, R164 within the core domain of NSs was identified as a key residue for the formation of NSs-dependent nuclear filaments in an alanine-scanning substitution experiment. RVFV-infected cells expressing an R164A variant of NSs displayed much higher levels of expression than the wild-type NSs protein. The synthesis of NSs mRNA depends on host transcription activity, and the higher expression level of the R164A variant of NSs in comparison to the wild-type protein suggests that this variant has a reduced capacity to inhibit host transcription as a result of a decreased capacity to form nuclear filaments. Additionally, levels of all subunits of TFIIH were higher with the R164A NSs variant, and this study demonstrated that formation of nuclear filaments by NSs correlates with degradation of several subunits of TFIIH [63].

In addition to R164, several other residues within NSs have been shown to be crucial for nuclear filament formation and inhibition of host transcription, including R16 and M250. Substitutions of these two residues were identified through serial passaging of MP-12, followed by plaque assay, and isolation of plaque-cloned viruses, to identify mutations in the viral genome that compensated for replication defects. Both an M250K and a double R16H/M250K substitution in NSs resulted in an increase in host transcription in RVFV-infected cells (Table 1) [68]. The NSs-R16H/M250K variant was unable to degrade p62 and inhibit general host transcription, but retained the ability to interact with SAP30, p62, and p44. In contrast, the NSs-M250K variant only slightly impaired the ability of NSs to inhibit general transcription. The ability of both variants of NSs to bind p44 and have a less pronounced inhibitory effect on host transcription inhibition emphasizes the importance of other TFIIH subunits, such as p62, for inhibition of cellular transcription, and indicates that the p62 subunit is more important for inhibition of cellular transcription than the p44 subunit. The mutants displayed reduced cytotoxicity and attenuated virulence in mice, emphasizing the importance of the NSs transcription-inhibition effect [68].

In addition to disrupting the assembly of TFIIH, NSs impacts host transcription on a genome-wide scale through interactions with the cellular chromatin machinery [82]. NSs does so through interactions with the promoter regions of 2786 genes. These interactions with the various promoter regions often resulted in transcriptional repression spanning innate immunity, inflammation, cell adhesion, axonal guidance, development, and coagulation. In particular, the host coagulation cascade was targeted through NSs interaction with the regulatory DNA regions of genes coding for multiple coagulation factors, including prothrombin, tissue factor (TF), or factors VII, VIII, and X, as well as the tissue factor pathway inhibitor (TFPI). In the case of TF, expression was repressed, whereas expression of prothrombin, factors VIII and X, and TFPI was upregulated. This varying regulation of multiple coagulation factors is significant in terms of disease progression following infection because RVFV-induced hemorrhagic fever is a leading cause of death in humans [83]. Taken together, it is clear that the NSs protein of RVFV is a global modulator of host gene expression through its ability to regulate host transcription at multiple levels, in addition to its well-documented degradation of multiple subunits of TFIIH [82].

### 4.3. Degradation of Protein Kinase R

The double-stranded RNA (dsRNA)-dependent protein kinase (PKR) plays an essential role in the host innate immune response following viral infection [84,85,86]. Upon sensing the presence of dsRNA produced during a viral infection, PKR phosphorylates eukaryotic initiation factor 2α (eIF2α), which results in a general shutdown of translation as a mechanism to prevent viral replication [87]. To counteract this and enable efficient viral translation, RVFV NSs induces the degradation of PKR, and this activity has been well documented [88,89,90]. NSs directs proteasome-dependent degradation of PKR through the NSs-SCF (Skp1, Cul1, F-box protein)-type E3 ubiquitin ligase complex, specifically through interaction with F-box proteins FBXW11 and β-TRCP1. Knockdown of Skp1 prevented PKR degradation and limited RVFV replication, demonstrating the importance of this complex in its ability to inhibit the innate immune response in infected cells [88,90,91].

To better understand the interaction between NSs and PKR, specific amino acid substitutions in NSs have been examined. It was determined that an R173A substitution in NSs (rMP12-NSsR173A) disrupts its ability to bind to PKR, which prevents the degradation of PKR (Table 1) [69]. In cells infected with the rMP12-NSsR173A variant of RVFV, PKR activates enhanced phosphorylation of eIF2α at early stages following infection, which contrasts with what is observed with the wild-type RVFV. In addition, viral proteins do not accumulate in rMP12-NSsR173A-infected cells, and the infected cells retain the ability to suppress host general transcription and IFN-β gene expression. Although the precise mechanism by which the R173A substitution leads to a loss of the PKR degradation function is unknown, this substitution does alter the morphology of the resulting NSs nuclear filaments [69].

## 5. Mitochondrial Modulation to Promote Inflammation

Inflammation is a critical component of the host defense mechanisms against viral infection [92,93], and mitochondria have recently emerged as key drivers of this mechanism [94]. The double-membraned mitochondria contain damage-associated molecular patterns (DAMPs) that trigger inflammatory responses when released into the cytosol via mitochondrial outer-membrane permeabilization (MOMP) [95]. MOMP is induced by activation of pore-forming, proapoptotic proteins BCL-2-associated X apoptosis regulator (BAX) and BCL-2 antagonist/killer 1 (BAK), leading to DAMP release into the cytosol and inflammatory response activation [96,97,98].

Recent studies have revealed a novel mechanism by which NSs promotes inflammation through modulation of the mitochondrial MCL-1-BAK axis [67]. More specifically, NSs was shown to promote inflammation by inducing transcriptional downregulation of the BAK inhibitor myeloid cell leukemia 1 (MCL-1). This downregulation leads to BAK activation, MOMP formation, mitochondrial reactive oxygen species (mtROS) accumulation, and subsequent release of oxidized mitochondrial DNA (ox-mtDNA) into the cytosol. The cytoplasmic ox-mtDNA then binds to the NLRP3 inflammasome, leading to NLRP3–gasdermin D (GSDMD) pyroptosis in RVFV-infected cells. Pyroptosis is a form of programmed cell death, and GSDMD is essential for both membrane pore formation and IL-1β secretion, which are mediated by caspase-1 following its recruitment to activated NLRP3 [99].

To investigate whether MCL-1 downregulation by NSs depends on its ability to inhibit transcription, the R16H/M250K variant of NSs was used (RVFV-NSs^RM^) (Table 1) [67,68]. In RVFV-NSs^RM^-infected cells, there was no downregulation of MCL-1 at either the protein or RNA level. This failure to downregulate MCL-1 expression was coupled with diminished BAK activation and reduced GSDMD cleavage, demonstrating impairment in pyroptotic signaling. Additionally, RVFV-NSs^RM^-infected cells had less mtDNA released into the cytosol and decreased IL1-β, mtROS, and mitochondrial damage. Collectively, these results indicate that NSs promotes BAK activation and triggers downstream pyroptosis through downregulation of MCL-1 by its host transcription-inhibition function [67].

In support of these in cellulo findings, in vivo studies utilizing both NLRP3 knockout mice and infection with either wild-type or RVFV-NSs^RM^ virus further demonstrated that NSs promotes inflammation through NLRP3 inflammasome activation. In both NLRP3 knockout mice and RVFV-NSs^RM^-infected mice, GSDMD cleavage, IL-1β release, liver inflammation, and fatality rate were reduced in comparison to wild-type. In RVFV-NSs^RM^-infected mice, NLRP3 inflammasome activation was not triggered. Viral loads across collected organs were similar. These in vivo studies support that NSs drives inflammation through NLRP3 activation rather than through increasing viral replication [67].

## 6. NSs Induction of the DNA Damage Response and Cell Cycle Arrest

The chemical integrity of cellular DNA is constantly under attack from exogenous and endogenous sources, resulting in the activation of the DNA damage response (DDR), a highly conserved pathway that monitors damage and maintains genome integrity [100]. When the DDR is activated, the cell cycle checkpoints arrest cells at either the G1/S, intra-S phase, or the G2/M boundaries [101]. These arrests allow the cell to either repair the DNA damage or activate apoptotic pathways leading to cell death [102]. RVFV infection has been shown to activate the DDR, specifically by activating ataxia telangiectasia mutated (ATM), leading to S phase arrest [103]. Phosphorylation of ATM and key DNA damage markers has been observed in RVFV-infected cells, including ATM (Ser-1981), p53 (Ser-15), Chk2 (Thr-68), and H2A.X (Ser-139). In contrast, neither DDR activation nor cell cycle arrest has been observed in cells infected with RVFV lacking NSs (RVFV ΔNSs), demonstrating NSs dependence in the induction of the DDR during RVFV infection [103] (Figure 4). Further, NSs induces characteristic effects of DNA damage through phosphorylation of H2A.X at serine 139, including formation of H2A.X foci in the nucleus, which leads to a positive feedback loop to enhance ATM phosphorylation and therefore the DDR. Additionally, it has been shown that RVFV NSs induces p53 phosphorylation at multiple serine residues and upregulation of p53 target genes involved in cell cycle arrest and apoptosis [104]. These studies indicate that NSs induces multiple phosphorylation events leading to induction of the DDR and cell cycle arrest.

When ATM and Chk.2 are inhibited, RVFV-induced cell cycle arrest was not observed, and RVFV replication was reduced, indicating the importance of these pathways for S phase arrest and viral replication. The importance of cell cycle arrest for RVFV replication can be at least partially explained by RVFV preferentially cap-snatching cell cycle mRNAs to support viral transcription [105,106]. Arrest in S phase increases the pool of cell cycle mRNAs and leads to more efficient RVFV replication [106].

Interestingly, the induction of ATM-dependent DDR does not appear to be dependent on increased DNA damage, as host DNA was not found to be damaged [103]. However, RVFV-infected cells have nuclear abnormalities, including chromosome cohesion and segregation defects, and this is reduced in RVFV Clone 13-infected cells, which encode a truncated NSs protein [107]. NSs was found to interact with pericentromeric DNA, which is DNA located near the centromeres and involved in chromosomal segregation. NSs Δ210–230, which is unable to interact with SAP30, did not interact with pericentromeric DNA or induce nuclear abnormalities [107]. Given these findings, it is possible that NSs interaction with pericentromeric DNA and the resultant nuclear abnormalities could trigger DDR signaling and cell cycle arrest, but additional studies are needed to more fully elucidate the events leading to DDR activation.

## 7. NSs Inhibits Autophagy

Autophagy is a conserved host pathway for maintaining cellular homeostasis that is often utilized by the immune system to clear out viruses and viral particles as part of the host antiviral response to infection [108,109]. In contrast, this system has also been shown to be hijacked by viruses such as RVFV to facilitate their replication and pathogenesis [70,110,111]. Following RVFV infection, autophagy induction has been observed in several cell types, but activation of autophagy by RVFV appears to be cell type-dependent [112,113]. Recently, the first mechanistic and structural insights into how NSs directly inhibits the host autophagy pathway through interaction with the host key autophagy protein family, the LC3-family members, were obtained [70].

More specifically, it was demonstrated that NSs contains four LC3-interacting region (LIR) motifs (NSs1, NSs2, NSs3, and NSs4), which are canonical motifs (W/F/Y-x-x-L/V/I) found in proteins that interact with the key autophagy protein LC3 [114]. However, it was determined that only the fourth LIR motif (NSs4: FVEV, residues 261–264), which is located in the intrinsically disordered C-terminus of the protein, was functionally important for regulating autophagy following infection with RVFV. It was determined that NSs interacts with all six human LC3 family members (LC3A, LC3B, LC3C, GABARAP, GABARAPL1, and GABARAPL2) via NSs4, and structural studies demonstrated that the first and fourth positions of NSs4 form multiple hydrophobic interactions with the LC3A and GABARAP proteins, indicating an essential role for these positions in NSs-LC3 interactions.

Under homeostatic conditions, LC3 proteins localize predominantly in the nucleus but are translocated to the cytosol, where LC3 performs its essential function in autophagosome formation upon induction of autophagy [115]. The NSs–LC3 interaction occurs primarily in the nuclear and perinuclear regions of cells, where LC3A was found to co-localize with NSs within the nuclear filaments [70]. This nuclear NSs–LC3 interaction prevents localization of LC3 to the cytosol and thus inhibits the host autophagy pathway by preventing autophagosome formation [70] (Figure 4). These results demonstrate that NSs interacts with LC3 via a LIR motif (NSs4), sequestering it in the nucleus and preventing it from performing its cytosolic autophagosome-formation function, thereby dampening the host autophagic response during infection [70].

To further investigate the importance of the LIR motif for NSs–LC3 interaction, cellular studies demonstrated that disruption of the NSs4 LIR motif through substitution of phenylalanine with serine at position 261 (F261S) significantly impaired NSs binding to all human LC3 family members (Table 1) [70]. This amino acid substitution restored the ability of LC3 to translocate to the cytosol and activated host autophagy function. There was also a significant increase in autophagic vesicle formation in RVFV NSs-F261S-infected cells when compared with wild-type RVFV MP-12-infected cells. Additionally, the F261S virus replicated less efficiently in immunocompetent cells than the wild-type virus but not in immunodeficient cells, demonstrating a link between autophagy and immune signaling. These results demonstrate that the F261 residue in NSs is crucial for NSs-LC3 binding and autophagy inhibition [70].

## 8. NSs Impacts Host Cytoskeleton Formation

The cytoskeleton is critical for maintaining cell morphology, facilitating motility, and coordinating the host immune response to the presence of pathogens, making it a key target for modulation by several viruses [116,117,118,119]. NSs affects the actin cytoskeleton of the host at both transcriptional and cellular levels [120]. At the transcriptional level, NSs expression suppresses the upregulation of Abl proto-oncogene 2 (Abl2) expression, a tyrosine kinase that plays a crucial role in regulating the actin cytoskeleton and cell motility [121,122]. Abl2 modulates cytoskeletal structure through regulation of Rho GTPases, and it has also been linked to the stabilization of adherens junctions via β-catenin. Adherens junctions are cell-to-cell adhesion structures that link the plasma membrane to the actin cytoskeleton [123,124]. NSs has been reported to disrupt adherens junctions, which weakens intercellular contacts and promotes tissue disintegration during infection [120]. Disruption of these adherens junctions can lead to enhanced viral spread and aid in the bypassing of innate immune barriers [120,121]. At the cellular level, NSs expression was associated with significant cellular morphology changes. These morphological changes resemble those seen in Abl2-deficient cells, including changes in cell shape, reduced spreading, and altered cellular adhesion [120].

The cytoskeletal changes are also attributed to direct interactions between NSs and the actin cytoskeleton. Actin is the primary component of the cytoskeleton, providing structural support, shape, and motility to eukaryotic cells [125]. In infected cells, NSs was found to be present within long, actin-rich intercellular structures, which are associated with its dissemination from NSs-expressing toward non-NSs-expressing cells [120]. These structures resemble those used by other viruses, such as influenza A and HIV-1, to promote cell-to-cell transmission and bypass immune detection [126,127]. The presence of NSs within these actin-rich intercellular connections suggests that RVFV hijacks the cellular cytoskeleton for both cytoskeletal remodeling and viral spread.

In addition to cytoskeletal remodeling, NSs significantly impacts host cell motility. Cells infected with a NSs deletion virus (RVFV ΔNSs) migrated significantly slower in comparison to cells infected with wild-type RVFV [120]. Given the association of Abl2 with reduced cell motility, it has been hypothesized that an upregulation of Abl2 may be a host mechanism to limit viral spread by restricting the migration of innate immune cells like macrophages, dendritic cells, neutrophils, and microglia [120,128]. Conversely, enhanced motility due to suppression of Abl2 activity in NSs-expressing infected cells may facilitate the viral spread and pathogenesis of RVFV [120].

Transcriptomic analyses of RVFV-infected cells further reinforced the impact of infection on the expression of cytoskeleton- and adhesion-related genes, including the integrin-linked kinase (ILK) pathway [129]. The ILK pathway is critical for cytoskeleton organization as it aids in cell migration, adhesion, and proliferation. In addition to Abl2, NSs downregulated gene expression of Lgals3bp, which encodes for an extracellular matrix protein promoting integrin-mediated cell–matrix and cell–cell adhesion, directly impacting the ILK pathway [130,131]. ILK connects the extracellular matrix to the actin cytoskeleton by interacting with the cytoplasmic tails of β integrins, regulating several functions related to actin cytoskeleton organization. The β3 integrin tail has been shown to directly interact with Abl2, connecting Abl2 with ILK signaling [121], and further supporting cell adhesion as a host cellular process targeted by RVFV.

Overall, these studies highlight the ability of NSs to modulate the host cytoskeleton by suppressing Abl2 expression, altering cellular morphology, disrupting adhesion structures such as adherens junctions, promoting actin-based intercellular extensions, and enhancing host cell motility.

## 9. NSs Blocks mRNA Export

Viruses block mRNA export as a strategy to suppress cellular translation and enhance viral replication [132,133]. A well-characterized example is the influenza A nonstructural protein 1, which localizes primarily in the nucleus and directly acts on mRNAs to inhibit host mRNA export [133,134]. During RVFV infection, NSs is responsible for blocking mRNA export [129,135,136]. The first indication that RVFV NSs may be influencing mRNA export was a study that showed NSs induced relocalization of the translation-related protein poly-adenylate binding protein 1 (PAB1) from the cytoplasm to the nucleus [136]. PAB1 binds to the 3’ poly-A tails of mRNA and interacts with eukaryotic initiation factor 4F (eIF4F), which binds 5’ mRNA caps. The PAB1–eIF4F interaction enhances ribosome initiation complex formation, thereby promoting translation. Nuclear sequestration of PAB1 inhibits translation, as PAB1 is exported via an mRNA-dependent export mechanism, and its nuclear accumulation alone is an indication that mRNA is blocked [136,137]. Indeed, mRNA export was found to be inhibited in RVFV-infected cells, as strong nuclear aggregates of polyadenylated RNA and a significant reduction in cytoplasmic mRNA signal were observed. This effect was not observed in cells infected with an NSs-deficient recombinant RVFV, directly implicating NSs in the mRNA export defect [135]. Moreover, NSs expression alone, in the absence of other viral components, was sufficient to reproduce this phenotype of nuclear mRNA accumulation [135]. When other viral proteins such as NSm, Gn, and N were expressed, the nuclear mRNA accumulation phenotype was not observed, demonstrating the critical role of NSs in blocking host mRNA export and its nuclear accumulation. The ability of NSs to block mRNA export was corroborated through a transcriptomics study in which a significant increase in gene expression following RVFV infection was observed without a corresponding increase in protein expression [129]. This disconnect between transcript abundance and protein output was attributed to NSs-mediated accumulation of mRNA in the nucleus [129]. While the exact mechanism by which NSs sequesters host mRNAs in the nucleus is still unknown, NSs-mediated inhibition of mRNA export is a key mechanism by which RVFV suppresses host gene expression and promotes viral replication.

## 10. NSs Manipulates Host Cell Apoptosis

Viral manipulation of host cell death pathways such as apoptosis is a well-studied mechanism that has been discussed in several reviews [138,139,140,141,142]. NSs has been shown to modulate the apoptosis pathway in a context-dependent manner, functioning in either a pro- or antiapoptotic fashion. Caspases are cysteine proteases that are a key component of the apoptotic process. Caspase-8 and -9 are considered initiator caspases, which are induced by apoptotic signals and activate the effector caspases through cleavage. In contrast, caspases-3, -6, and -7 are considered effector caspases that are responsible for cleaving cellular proteins and executing the apoptotic cascade [143]. During RVFV infection, intranuclear co-localization of the NSs protein and active caspase-3 was observed in hepatocytes of immunocompetent hzNU mice infected with the virulent RVFV 35/74 strain, but not in attenuated MP-12-infected IFNAR-/- mice [144]. This NSs-activated caspase-3 co-localization is associated with the formation of intranuclear inclusion bodies, which are a known component of severe RVFV-induced hepatitis. The nuclear filaments that are immunoreactive with active caspase-3 in the RVFV 35/74-infected livers deviate from the typical granular nuclear distribution of active caspase-3 in apoptotic cells, suggesting a possible mechanism for delayed apoptosis that may enhance viral replication via NSs. There is a strain- and cell type-dependent role of NSs in modulating apoptosis, as demonstrated by the differences in the RVFV MP-12-infected vs. RVFV 35/74-infected mice. NSs intranuclear recruitment of active caspase-3 contributes to both the pathogenesis and distinct histopathology of virulent RVFV infection [144].

Another mechanism by which NSs modulates apoptosis is through NSs-dependent phosphorylation of the transcriptional regulator signal transducer and activator of transcription 3 (STAT3) at a conserved tyrosine residue (Y705) [60]. STAT proteins are a family of transcription factors that regulate host cell processes, including immunity, survival, and proliferation [145]. During the Jak-STAT protein cascade, STAT proteins are phosphorylated, leading to nuclear translocation. Phosphorylation of STAT3 was significantly increased following RVFV infection, but little to no phosphorylation was observed in cells infected with the RVFV ΔNSs variant, demonstrating the crucial role of NSs for the increase in STAT3 phosphorylation during infection. Loss of STAT3 led to an increase in cell susceptibility to RVFV-induced cell death, indicating a pro-survival role for STAT3 during RVFV infection. The translocation of phosphorylated STAT3 to the nucleus was associated with reduced expression of several proapoptotic genes, including the nuclear receptor subfamily 4 group A member 2 (nr4a2). STAT3 and NSs were found to co-localize at the nr4a2 promoter, and this correlated with an increase in K9 methylation of histone H3 (H3K9Me3), and is consistent with NSs and STAT3 transcriptionally repressing nr4a2. These findings demonstrate a role for NSs in inhibiting apoptosis through STAT3 by suppressing proapoptotic gene expression [60].

Reactive oxygen species (ROS) are critical host components that contribute to innate immune response activation and directly influence the clearance of pathogens [146]. Following RVFV infection, ROS levels are significantly increased in liver cells. The observed increase is dependent on the expression of NSs, which localizes to the mitochondria, driving oxidative stress and resulting in activation of proapoptotic responses including p53 and NF-kB (via its p65 subunit) [147]. Following RVFV infection, p53 is phosphorylated on serine 15, 46, 9 20, 37, and 392 following expression of NSs, which results in an increase in total p53 protein levels, nuclear accumulation of p53, and the induction of the p53-regulated proapoptotic gene, Noxa [104]. Cells lacking p53 demonstrated reduced viral titers and less RVFV-induced cell death, suggesting that p53 activation aids in efficient viral release and dissemination through the promotion of the apoptotic response [104]. In parallel, RVFV also triggers activation and nuclear localization of NF-κB during early stages of infection, demonstrating that NSs promotes activation of the proapoptotic p65 and p53 pathways in liver cells. Viral protein-mediated disruption of mitochondria results in increased intracellular ROS and oxidative stress due to disruption of the mitochondrial electron transport machinery [148,149]. Given this, it is tempting to speculate that NSs, through its mitochondrial association, could influence intracellular ROS levels at early stages of an RVFV infection by disrupting the electron transport chain due to ion imbalances or calcium influx into the mitochondria. Additionally, treatment of RVFV-infected cells with antioxidants such as curcumin significantly reduced levels of ROS, activation of p53, induction of apoptotic gene expression, and apoptosis [147]. Taken together, these findings demonstrate that NSs manipulates the host apoptotic pathways by targeting several different factors, allowing it to either enhance or delay apoptosis in a strain- and cell-dependent context.

## 11. NSs Regulates the Induction of Smad Signaling

Sma- and Mad-related proteins (Smad1, 2, 3, 5, and 8/9) are evolutionarily conserved transcription factors that play a key role in regulating the transforming growth factor-beta (TGF-β) superfamily signaling pathways and thereby mediate outcomes during embryogenesis, inflammation, and immunity [150]. There is extensive literature suggesting Smad proteins are prominent targets during many viral infections [151,152,153,154,155]. Smad proteins are both phosphorylated by and activated by TGF-β superfamily receptors [156] and high levels of phosphorylation of Smad1/5/9 and Smad 2 transcription factors have been observed following infection with either the BSL-2 or BSL-3 strains of RVFV [157]. Phosphorylation of these Smad proteins requires active RVFV replication, and increased Smad protein phosphorylation was not observed in RVFV ΔNSs-infected cells. Interestingly, NSs expression alone was insufficient to induce Smad phosphorylation, but its presence during viral replication was crucial for Smad phosphorylation. Knockdown of Smad proteins did not impact viral replication, but it is thought that Smad activation may have a direct contribution to pathogenesis [157]. One Smad target identified through promoter analysis was interleukin-1 receptor type 2 (IL1R2), a cytokine receptor that acts as a decoy for interleukin-1 (IL-1) family cytokines, thereby reducing proinflammatory IL-1 signaling. Chromatin immunoprecipitation demonstrated enrichment of Smad4-containing complexes on the IL1R2 promoter after RVFV infection, demonstrating that Smad signaling downstream of NSs could lead to an immunosuppressive environment. In addition, a report with human cases of RVFV infection demonstrated that in fatal cases there is an increase in interleukin-10 and interleukin-1 receptor antagonist (IL1RA), both of which are immunosuppressive, demonstrating that the host response during infection often shifts towards suppression rather than inflammation [158]. Both IL1RA and IL1R2 inhibit IL-1 signaling, and their upregulation during infection dampens the innate immune response, allowing the virus to evade early detection. The activation of Smad protein phosphorylation and promotion of decoy receptors by NSs may play a role in shifting the host immune response to suppression, thereby allowing NSs to enhance viral pathogenesis [157].

## 12. Conclusions and Future Perspectives

RVFV is a devastating pathogen to livestock and humans alike [159]. Currently found only in Africa and the Arabian Peninsula, the virus continues to cause fatal outbreaks. Alarmingly, there is a significant threat for the global spread of RVFV, as competent mosquito vectors have been identified in both the United States and Europe [21,22]. NSs is the main virulence factor of RVFV, making it an attractive therapeutic target, as it acts by hijacking several host cellular processes to evade the host innate immune response. This review summarizes our current understanding of the different mechanisms by which NSs helps RVFV evade the host immune system during infection, including the impact of specific amino acid substitutions in NSs that functionally disrupt several of the discussed viral takeover mechanisms.

Despite extensive research on the ability of NSs to inhibit host transcription and induce PKR degradation, there are several underexplored areas for understanding the different functions carried out by NSs that could provide more biological insight into how this viral Houdini acts to take over the cell. Several variants of RVFV containing modified versions of NSs have been well studied to understand the mechanistic role of NSs in inhibiting transcription and forming nuclear filaments. However, there is still a significant knowledge gap in understanding which regions of NSs are required for modulating pathways such as the host DNA damage response, apoptosis, and the Smad signaling pathway. In addition, there are few NSs–host interacting partners identified to date. Mass spectrometry-based interactome analysis could help address the latter knowledge gap. There is one published NSs interactome study that identified a few NSs-interacting partners such as FBX03, Nup98, and Rae1 [59]. However, additional global characterization of the NSs interactome through newer approaches could provide insights into protein–protein interactions needed to regulate these pathways, as well as uncover additional host processes mediated by NSs. For example, using proximity-dependent biotinylation (Bio-ID) coupled with mass spectrometry could enable identification of transient proteins such as kinases [160,161], which are difficult to detect with standard immunoprecipitation methods. Given that NSs interacts with multiple E3 ubiquitin ligases, it is likely that NSs mediates the degradation of yet-to-be-identified host proteins, and interactome analysis in the presence of proteasome inhibitors could uncover novel targets.

Finally, much of the research on RVFV NSs is performed using RVFV MP12, which is a live attenuated vaccine strain. NSs from MP12 is identical at the amino acid level to its parental virulent strain ZH548, making it a good surrogate to study NSs functions. However, it is still important to verify that the pathways impacted during RVFV MP12 infection are relevant to infection with virulent strains of RVFV. This is particularly important as pathways modulated by RVFV NSs may be considered as targets for host-based therapeutics and their contribution to pathogenesis should be confirmed.

## Data Availability

No new data were created or analyzed in this study. Data sharing is not applicable to this article.
